
JOURNAL INFORMATION
==============================
NLM Title Abbreviation: ScientificWorldJournal
Journal ID: ScientificWorldJournal
NLM Journal ID: 101131163
Journal ID: TSWJ

The Scientific World Journal

ISSN: 2356-6140
EISSN: 1537-744X
Publisher: Wiley

ARTICLE INFORMATION
==============================
PMCID: PMC6084524
PMID: 30147471
DOI: 10.1100/tsw.2001.158
Article ID: 915091
Article version: 1
Subjects: Short Report

Nitric Oxide Protects Mouse Thymocytes from Apoptosis Induced by γ-Irradiation

Chen Yue *
Stanford Ala
Hoffman Rosie
Ford Henri
Department of Surgery, University of Pittsburgh, School of Medicine, and the Children’s Hospital of Pittsburgh, 3705 Fifth Avenue, Pittsburgh, PA 15213, USA
*Yue Chen: cheny2@msx.upmc.edu
Publication date: 2001
Electronic publication date: 2001 Nov 20
Volume: 1
First page: 34
Last page: 34
Copyright: Copyright © 2001 Yue Chen et al.
Copyright year: 2001
License: This is an open access article distributed under the Creative Commons Attribution License, which permits unrestricted use, distribution, and reproduction in any medium, provided the original work is properly cited.
License URL: https://creativecommons.org/licenses/by/3.0/

==============================
Miami *Nature Biotechnology* Short Reports
*TheScientificWorld* (2001) 1(S3), 34SR
ISSN 1532-2246; DOI 10.1100/tsw.2001.158

### NITRIC OXIDE PROTECTS MOUSE THYMOCYTES FROM APOPTOSIS INDUCED BY γ-IRRADIATION

Yue Chen*, Ala Stanford, Rosie Hoffman, and Henri Ford

Department of Surgery, University of Pittsburgh, School of Medicine, and the Children’s Hospital of Pittsburgh, 3705 Fifth Avenue, Pittsburgh, PA 15213
* cheny2@msx.upmc.edu

**INTRODUCTION.** Nitric Oxide (NO) and its reactive products can either promote or prevent apoptosis depending upon the cell systems and conditions involved (1). We have previously reported that NO-induced mouse thymocyte apoptosis in vitro involves p53 upregulation and caspase-1 activation (2, 3). To further dissect the relationship between NO and thymocyte apoptosis, we investigated the effect of NO on Balb/c thymocytes exposed to γ-irradiation. We found that NO partially protects γ-irradiated thymocytes from apoptosis by preserving mitochondrial integrity and inhibiting caspase activity.

**METHODS.** A single cell suspension was prepared from freshly isolated Balb/c thymus. Thymocytes were exposed to γ-irradiation in the presence or absence of an exogenous NO donor, S-nitroso-N-acetyl penicillamine (SNAP), for various time intervals. Thymocyte apoptosis was detected by Annexin V/PI staining followed by flow cytometry. Mitochondrial transmembrane potential was measured using the fluorescent dye, DiOC6, and flow cytometry. Western Blot was performed on thymocyte lysates. Caspase activity in cell lysates was measured by a colorimetric assay using specific substrates.

**RESULTS**. The percentage of apoptotic thymocytes at 4 and 8 hours following γ-irradiation (5Gy) was significantly decreased in the presence of 1 mM SNAP, compared to γ-irradiation alone (see Fig. 1). γ-irradiation induced release of cytochrome c and reduction in mitochondrial transmembrane potential, which were inhibited by SNAP (see Fig. 2). The increased activities of caspase 3, 6, 8 and 9 at 6 hours following γ-irradiation were diminished by SNAP (see Fig. 3). The cleavage of caspase 3, 6, and PARP in γ-irradiated cells was inhibited by co-incubation with SNAP at 6 hour post γ-irradiation (see Fig. 4).

**Fig. 1**

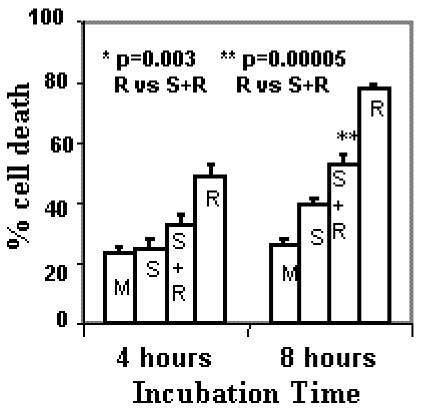

M: medium, S: SNAP,
S+R: SNAP+Radiation, R: Radiation

**Fig. 2**

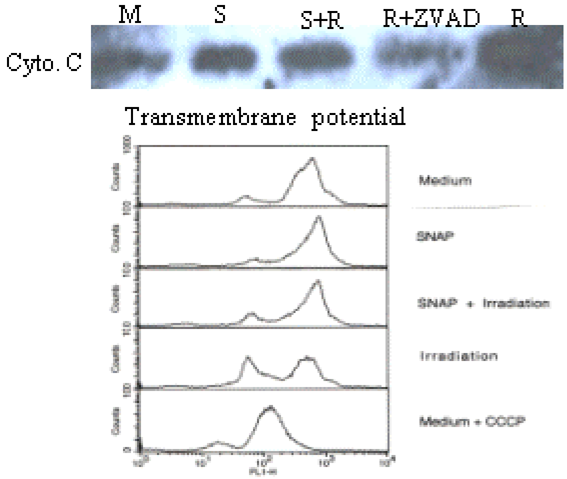

**Fig. 3**

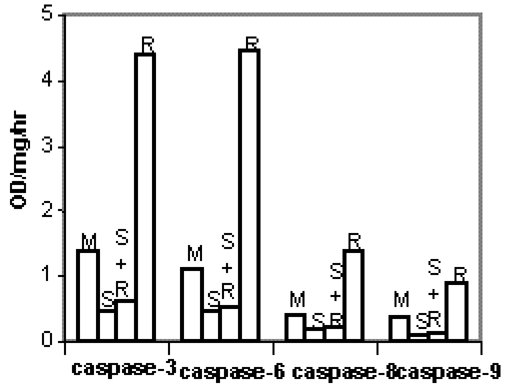

**Fig. 4**

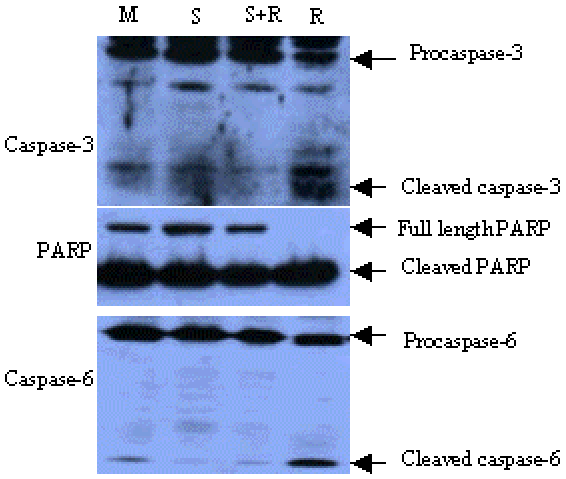

**DISCUSSION**. γ-irradiation induces rapid apoptosis in Balb/c thymocytes by activating apoptotic signaling pathways (4). The anti-apoptotic activity of NO has been documented in certain cell types (1). Our results show that NO partially inhibits γ-irradiation-induced thymocyte apoptosis by decreasing mitochondrial damage and suppressing caspase activity. Western Blot showed that the cleavage of caspase-3 and caspase-6 was inhibited by SNAP. The data suggest that NO may act on upstream molecules in the apoptotic pathway to inhibit apoptosis. The detailed mechanism of this protective effect is under intense investigation at present time.

**REFERENCES.**

1. Kim, Y.M., Bombeck, C.A., and Billiar, T.R. (1999) Circ. Res. 84, 253-256
2. Zhou, X. et al. (2000) J. Immunol. 163, 1252-1258
3. Gordon, S.A. et al. (submitted for publication)
4. Yang, Y. and Ashwell, J.D. (1999) J. Clin. Immunol. 19, 337-349
